# Estimation of Physical Activity Levels Using Cell Phone Questionnaires: A Comparison With Accelerometry for Evaluation of Between-Subject and Within-Subject Variations

**DOI:** 10.2196/jmir.1686

**Published:** 2011-09-25

**Authors:** Christin Bexelius, Sven Sandin, Ylva Trolle Lagerros, Jan-Eric Litton, Marie Löf

**Affiliations:** ^1^Department of Medical Epidemiology and Biostatistics, Karolinska InstitutetStockholmSweden; ^2^Unit of Clinical EpidemiologyDepartment of Medicine, Karolinska InstitutetStockholmSweden; ^3^Department of Clinical and Experimental Medicine, University of LinköpingLinköpingSweden

**Keywords:** Cell phone, Internet, physical activity, epidemiology

## Abstract

**Background:**

Physical activity promotes health and longevity. Further elaboration of the role of physical activity for human health in epidemiological studies on large samples requires accurate methods that are easy to use, cheap, and possible to repeat. The use of telecommunication technologies such as cell phones is highly interesting in this respect. In an earlier report, we showed that physical activity level (PAL) assessed using a cell phone procedure agreed well with corresponding estimates obtained using the doubly labeled water method. However, our earlier study indicated high within-subject variation in relation to between-subject variations in PAL using cell phones, but we could not assess if this was a true variation of PAL or an artifact of the cell phone technique.

**Objective:**

Our objective was to compare within- and between-subject variations in PAL by means of cell phones with corresponding estimates using an accelerometer. In addition, we compared the agreement of daily PAL values obtained using the cell phone questionnaire with corresponding data obtained using an accelerometer.

**Methods:**

PAL was measured both with the cell phone questionnaire and with a triaxial accelerometer daily during a 2-week study period in 21 healthy Swedish women (20 to 45 years of age and BMI from 17.7 kg/m^2^ to 33.6 kg/m^2^). The results were evaluated by fitting linear mixed effect models and descriptive statistics and graphs.

**Results:**

With the accelerometer, 57% (95% confidence interval [CI] 40%-66%) of the variation was within subjects, while with the cell phone, within-subject variation was 76% (95% CI 59%-83%). The day-to-day variations in PAL observed using the cell phone questions agreed well with the corresponding accelerometer results.

**Conclusions:**

Both the cell phone questionnaire and the accelerometer showed high within-subject variations. Furthermore, day-to-day variations in PAL within subjects assessed using the cell phone agreed well with corresponding accelerometer values. Consequently, our cell phone questionnaire is a promising tool for assessing levels of physical activity. The tool may be useful for large-scale prospective studies.

## Introduction

Large epidemiological studies on physical activity and health require accurate methods that are easy to use, cheap, and possible to repeat. Recently, the potential of using cell phones, either through short message service (SMS) or Web-like applications, in behavior change intervention studies have been explored (for example, [1-4]). However, cell phones also open new possibilities for data collection in large-scaled prospective studies [5,6].

When assessing physical activity, one important aspect is the total amount of energy expended due to physical activity. This variable can be obtained as total energy expenditure divided by basal metabolic rate, that is, the so-called physical activity level (PAL) [7]. We have recently developed a Java-based cell phone questionnaire to assess PAL that places little demand on either the study center or the participants [8]. Once every day for 2 weeks subjects are asked two questions via their cell phones about their daily physical activity. In an earlier study, the mean PAL during 2 weeks using this cell phone questionnaire agreed well with corresponding estimates based on the doubly labeled water method and indirect calorimetry (mean difference = 0.014, 1 standard deviation [SD] = 0.15) [8]. However, our earlier study showed a low variation in PAL between subjects (20% of the total variation) [8], indicating that the rest (80%) was caused by variations within subjects. Since the doubly labeled water method does not provide daily PAL values, we could not evaluate whether these variations were true or not. If they are not true, a low between-subject variation in our cell phone questionnaire may indicate that these estimates are not able to distinguish PAL between individuals very well. Thus, the aim of this study was to compare within- and between-subject variations in PAL by means of cell phones with corresponding estimates using an accelerometer. In addition, we compared the agreement of daily PAL values obtained using the cell phone questionnaire with corresponding data obtained using an accelerometer.

## Methods

In all, 22 healthy nonsmoking Swedish women were recruited during August 2007 through May 2008 as previously described [8]. The women were 35.1 (range 20-45, SD 8.3) years of age, and their BMI was 23.7 kg/m^2^ (range 17.7 kg/m^2^ to 33.6 kg/m^2^, SD 3.8 kg/m^2^). PAL was measured daily during a 14-day study period using the cell phone questions as well as a tri-axial accelerometer, the RT3 (Stayhealthy Inc, Monrovia, CA, USA). The first day of the study period was excluded since the women did not wear the accelerometer until the afternoon that day; thus, results are reported for 13 days. One woman accidently broke her accelerometer; thus, results are reported for only 21 women. The study was approved by the central ethics board in Stockholm, Sweden.

Our JAVA-based questionnaire for assessing PAL using cell phones has previously been described in detail [8]. Briefly, at 9 pm, each woman was asked two questions about her physical activity during the same day (Table 1). For each woman and for each day, the answers to the two short questions were converted to PAL by adding the PAL points obtained for work/day time activities [9] and the PAL points obtained for leisure/evening activities [10] (Table 1).

**Table 1:** The cell phone questionnaire consisting of two questions

Cell Phone Question		PAL Points
**Question 1: How physically active have you been during work/the daytime today?**			
	Mostly sitting	1.55
	Sitting/standing/walking	1.65
	Standing/walking most of the time	1.85
	Heavy work	2.2	
**Question 2: How physically active have you been during leisure time/the evening today?**			
	Mostly sitting	+0
	Light/walking 30 minutes	+0.06
	Moderate/cycling ≥ 30 minutes	+0.15
	Sport/cycling ≥ 60 minutes	+0.29

Each woman was instructed to wear the accelerometer during all her waking hours except when in water. She recorded in a notebook when she took off the device and the activities performed without it (eg, showering or sleeping). Recorded movements were converted to total energy expenditure according to the manufacturer. All women wore the RT3 all 14 days and the recordings obtained during these days covered 97% ± 2% of time awake. For activities reported in the notebook, energy expenditure was estimated based on published energy costs of specific activities [10]. PAL was obtained as total energy expenditure divided by resting metabolic rate (calculated using in-built equations provided by the RT3 manufacturer) for each 24-hour period during days 1 to 13 (starting at 9 pm the first study day).

Intraclass correlation coefficients (ICC) were calculated as the between-subject variation divided by the total variation.The coefficients were estimated by means ofa linear mixed effect model with the two components of variance and a fixed effect for days 1 to 13. The ICC was used to compare the between-subject variation in relation to total variation for PAL values at days 1 to 13. The ICC was calculated from models fitted separately for the cell phone data and the accelerometer data. To test the robustness against the influence of single gross outliers, we repeated the ICC calculations for both methods when we had replaced the 10 as well as 20 most deviating daily PAL values out of 273 values with the mean PAL for these women. Furthermore, the agreement of daily PAL values obtained using the cell phone questionnaire and corresponding accelerometer data was evaluated using descriptive statistics. All statistical analyses were carried out using the SAS software proc mixed, version 9.2 (SAS Institute, Inc, Cary, NC, USA).

## Results

For PAL obtained using the cell phone, the variance between and within subjects was estimated to be 0.009 and 0.029, respectively. Thus, 24% of the total variation was between subjects, and 76%, within subjects. The corresponding estimates for the accelerometer were 0.017 and 0.023, showing that 43% of the variation was between subjects and 57%, within subjects. The 95% CI for the within-subject variation was 59% to 83 % for the cell phone while it was 40% to 66% for the accelerometer. The variance components for PAL obtained using the cell phone and the accelerometer remained similar when we replaced the 10 and 20 most deviating values with the mean PAL of these women.

**Figure 1:**
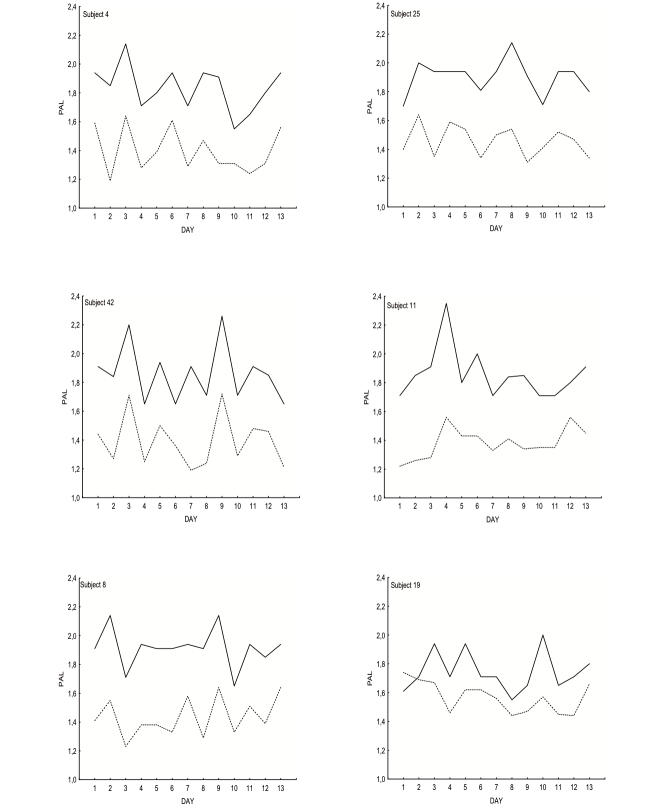
Daily measures of PAL over a 13-day period for 6 randomly chosen individuals (the dotted line indicates accelerometer data and the straight line indicates PAL data from cell phones).

Figure 1 compares daily PAL values obtained with cell phones and accelerometers from day 1 to day 13 for 6 randomly selected subjects. Daily PAL values obtained with the accelerometer were generally on a lower level than the corresponding cell phone estimates, but the day-to-day changes in PAL observed using the cell phone questions followed the corresponding changes in the accelerometer PAL. Similar agreement between daily PAL values obtained using the two methods was observed for the other 15 women (data not shown).

## Discussion

Both the cell phone questionnaire and the accelerometer showed high within-subject variations, indicating that there is considerable true day-to-day variation in PAL. First, this makes it more likely that the relative low between-subject variations we reported in our earlier paper are not artifacts of the cell phone technique. Second, this is an interesting finding since few reports have described the specific sources of variance in daily physical activity using objective measures in adults [11-13].

PAL obtained using the accelerometer was generally on a lower level than the cell phone value. The RT3 as well as several other accelerometers have shown a tendency to underestimate energy expenditure during free-living conditions compared with the doubly labeled water method [14-15] also in the women in this study (Löf et al, unpublished data). Since the changes in daily PAL values obtained using the RT3 accelerometer agreed well with the corresponding cell phone estimates, we hypothesize that the underestimation in PAL results from an underestimation of energy expenditure from movements. Future studies should explore why the RT3 underestimates energy expenditure and how the monitor may be refined in order to improve its predictions.

With the accelerometer, 43% of the variation in PAL was between subjects, while between-subject variation was 24% with the cell phone. For comparison, Matthews et al [12] reported that differences between subjects accounted for 55% to 60% of the variation in accelerometer counts in 92 healthy adults, but their study covered a wider age range (18 to 79 years of age) than our study. For a paper questionnaire, the between-subject variation in overall physical activity was 20% to 30% in 580 healthy adults [16]. As discussed by Matthews et al, the variance structure between self-reported and objective measures may differ due to different inherent errors [12]. A limitation of accelerometers is that they are not sensitive to activities involving upper body movements such as weight-lifting or carrying. Weight-lifting was not common, but we have no information about carrying. If carrying was evenly distributed among our women, the variance components would be unchanged, but if carrying was unevenly distributed, the true between-subject variation using the accelerometer may be even higher. Thus, the results likely indicate that the cell phones underestimate the between-subject variation. One plausible explanation is the answer options. The highest category for the second question only mentions sports and cycling, while home chores like gardening are not included. Such activities could be added to refine the capability of the cell phone questionnaire to assess between-subject variation in PAL.

The major limitation of this study is the small sample size. First, it makes our analysis sensitive to single gross outliers since these may largely increase the within-subject variations. However, we found no evidence for any important effect of such outliers in our analysis since the variance components were stable when we replaced the 10 or 20 most deviating PAL values (out of totally 273) with the average PAL for those women. Second, the small sample size limits our ability to make firm conclusions about the between-subject variations. We cannot exclude that the low between-subject variation obtained by both methods to some extent is due to the fact that our participants were a relatively small group of women. Other limitations are that we estimated energy expenditure for activities when the women were not wearing the RT3, but this amount of time was small, and that this study was conducted in healthy, moderately active women. The results should be repeated in other populations including men as well as elderly and obese subjects.

In conclusion, (1) Both the cell phone questionnaire and the accelerometer showed high within-subject variations in PAL and (2) changes in daily cell phone PAL values agreed well with corresponding accelerometer values. This study adds further evidence to our earlier findings [8] that the cell phone questionnaire is a promising tool for assessing PAL in epidemiological studies.

## Abbreviations

CI: confidence interval
ICC: Intraclass correlation coefficient
PAL: physical activity level
SMS: Short Message Service
